# Vegan Diet and Bone Health—Results from the Cross-Sectional RBVD Study

**DOI:** 10.3390/nu13020685

**Published:** 2021-02-21

**Authors:** Juliane Menzel, Klaus Abraham, Gabriele I. Stangl, Per Magne Ueland, Rima Obeid, Matthias B. Schulze, Isabelle Herter-Aeberli, Tanja Schwerdtle, Cornelia Weikert

**Affiliations:** 1 Department of Food Safety, German Federal Institute for Risk Assessment, 10589 Berlin, Germany; klaus.abraham@bfr.bund.de (K.A.); cornelia.weikert@bfr.bund.de (C.W.); 2 Institute for Social Medicine, Epidemiology and Health Economics, Charité, Universitätsmedizin Berlin, 10117 Berlin, Germany; 3 Institute for Agricultural and Nutritional Sciences, Martin Luther University Halle-Wittenberg, 06120 Halle (Saale), Germany; gabriele.stangl@landw.uni-halle.de; 4 Section for Pharmacology, Department of Clinical Science, University of Bergen, 5021 Bergen, Norway; per.ueland@ikb.uib.no; 5 Department of Clinical Chemistry and Laboratory Medicine, Saarland University Hospital, 66421 Homburg, Germany; rima.obeid@uks.eu; 6 Department of Molecular Epidemiology, German Institute of Human Nutrition Potsdam–Rehbruecke, 14558 Nuthetal, Germany; mschulze@dife.de; 7 Institute of Nutritional Science, University of Potsdam, 14558 Nuthetal, Germany; 8 Laboratory of Human Nutrition, Institute of Food, Nutrition and Health, ETH Zurich, 8092 Zurich, Switzerland; isabelle.herter@hest.ethz.ch; 9 German Federal Institute for Risk Assessment, 10589 Berlin, Germany; tanja.schwerdtle@bfr.bund.de; 10 Department of Food Chemistry, Institute of Nutritional Science, University of Potsdam, 14558 Nuthetal, Germany

**Keywords:** bone health, BUA, SOS, QUS, vegan, diet, biomarker, reduced rank regression, RRR

## Abstract

Scientific evidence suggests that a vegan diet might be associated with impaired bone health. Therefore, a cross-sectional study (*n* = 36 vegans, *n* = 36 omnivores) was used to investigate the associations of veganism with calcaneal quantitative ultrasound (QUS) measurements, along with the investigation of differences in the concentrations of nutrition- and bone-related biomarkers between vegans and omnivores. This study revealed lower levels in the QUS parameters in vegans compared to omnivores, e.g., broadband ultrasound attenuation (vegans: 111.8 ± 10.7 dB/MHz, omnivores: 118.0 ± 10.8 dB/MHz, *p* = 0.02). Vegans had lower levels of vitamin A, B2, lysine, zinc, selenoprotein P, n-3 fatty acids, urinary iodine, and calcium levels, while the concentrations of vitamin K1, folate, and glutamine were higher in vegans compared to omnivores. Applying a reduced rank regression, 12 out of the 28 biomarkers were identified to contribute most to bone health, i.e., lysine, urinary iodine, thyroid-stimulating hormone, selenoprotein P, vitamin A, leucine, α-klotho, n-3 fatty acids, urinary calcium/magnesium, vitamin B6, and FGF23. All QUS parameters increased across the tertiles of the pattern score. The study provides evidence of lower bone health in vegans compared to omnivores, additionally revealing a combination of nutrition-related biomarkers, which may contribute to bone health. Further studies are needed to confirm these findings.

## 1. Introduction

In recent years, plant-based diets have become increasingly popular in Germany and many other Western countries [1,2]. In particular, a growing trend toward a vegan diet has been observed, referring to a diet without consumption of any animal products. People are turning to a vegan diet not only due to compassion for animals and awareness of environmental problems but also for health benefits [1]. Indeed, scientific evidence suggests that a vegan or vegetarian diet may protect against many chronic diseases, e.g., diabetes [3], cardiovascular diseases [4], or cancer [5]. However, a vegan diet was found to be associated with lower bone mineral density (BMD), which is associated with higher fracture risk, compared to omnivores [6]. The skeleton is a dynamic and metabolically active tissue [7] and is exquisitely sensitive to its microenvironment [8]. Accordingly, nutritional habits have been considered an important modifiable factor influencing BMD [8,9]. Consuming a vegan diet arises concern about an inadequate supply of some nutrients [10], possibly contributing to an impaired BMD in vegans. For instance, calcium and vitamin D are well known as major determinants of bone health [9], but they are considered as potential critical nutrients in vegans [10]. Other critical nutrients for a vegan diet are long-chain n-3 fatty acids [10], vitamins (B12, A) [10], or minerals (zinc, selenium, iodine) [10], which are also related to bone health [8]. On the other hand, vegetarian and vegan diets provide important nutrients that protect bone, e.g., vitamin K [7,8,11] and folate [7,12,13,14].

Therefore, the present study aimed to investigate the differences in bone health between vegans and omnivores, as measured using quantitative ultrasound. Furthermore, the study aimed to detect differences in nutritional biomarkers that are related to bone health (selected vitamins, minerals, fatty acids, and amino acids), along with differences in biomarkers of bone turnover, calcium homeostasis, inflammation, and the fibroblast growth factor 23 (FGF23)–α-klotho axis. In addition, via the application of reduced rank regression (RRR), the study aimed to detect an exploratory biomarker pattern that may reveal a combination of biomarkers that contribute to bone health and thereby may explain the suggested reduced bone health in vegans. As is known, classic endocrine feedback loops ensure the regulation of blood calcium alongside the involvement of parathyroid hormone (PTH), vitamin D, and FGF23 [15] having their own impacts on bone health; thus, the complexity of the homeostatic regulatory biomarkers of bones should be considered, too. Therefore, the RRR included not only classical nutritional biomarkers but also other important nutrition-associated bone-related biomarkers.

## 2. Materials and Methods

### 2.1. Study Population 

The study participants were investigated between January 2017 and July 2017. Participants of the present “Risks and Benefits of a Vegan Diet” (RBVD) study were individuals who responded to an advertisement by contacting the study center at the German Federal Institute for Risk Assessment (BfR) via phone or mail (*n* = 161). A phone screening followed, including a brief explanation of the study and checking the inclusion criteria (age 30–60 years, following the diet for at least 1 year) and exclusion criteria (body mass index (BMI) ≥ 30, cardiovascular disease, type 2 diabetes, cancer, pregnancy, breastfeeding, current infection) [16]. An omnivorous diet was defined as the consumption of at least three portions of meat per week or two portions of meat and two portions of processed meat per week, whereas a vegan diet was defined as no consumption of any animal food products [16]. The cross-sectional study was conducted at the BfR in Berlin, Germany. Each participant visited the study center twice [16]. On their first visit, participants gave their written informed consent and received instructions to collect 24 h urine and to document their diet using a three-day weighed food protocol. At the second visit, anthropometric measurements, a quantitative ultrasound measurement, and their lifestyle characteristics were assessed, and a fasting blood sample was collected [16]. In total, the final study population comprised 36 vegans and 36 omnivores that were sex- and age-matched. A flowchart was published previously [16]. The study was approved by the Ethics Committee of Charité University Medical Center Berlin (no. EA4/121/16) and was conducted in accordance with the Declaration of Helsinki.

### 2.2. Quantitative Ultrasound Measurement

In our study, bone health was assessed using quantitative ultrasound (QUS) measurements. According to the manufacturer’s instructions, QUS measurements were performed by trained personnel on the right and left os calcis using the Achilles EXPII bone ultrasonometer (General Electric Healthcare, Little Chalfont, UK). In the case of unilateral foot pathology (ankle edema, trauma, or fracture) of a heel, only the opposite heel was measured. The instrument measures the frequency-dependent broadband ultrasound attenuation (BUA) (dB/MHz) and the speed of sound (SOS) (m/s). The stiffness index (SI) was automatically calculated from the BUA and SOS using the Achilles EXPII system via the following equation: stiffness index = (0.67 × BUA + 0.28 × SOS) − 420 [17]. The mean values of quantitative ultrasound measurements were calculated from the left and right heel measurements, except in four participants, where only one heel site measurement was available. Due to the anatomical conditions of the feet, the measurement for one participant was not possible. 

### 2.3. Assessment of Lifestyle Characteristics

Anthropometric measurements (weight, height, and waist circumference) were performed by trained and quality-monitored personnel while the participants wore only light underwear and had no shoes on. Body weight was assessed using an electronic digital scale (Omron BF511 Omron Healthcare Ltd., Kyoto, Japan) and the height was measured using a flexible anthropometer (SECA 213, Hamburg, Germany). Waist circumference was defined using the horizontal plane midway between the lowest ribs and the iliac crest. Information on educational level, smoking habits, and supplement intake was assessed using computer-based questionnaires. The educational levels were defined as high education (university, university of applied sciences), intermediate education (vocational school, technical college), or low education (no degree). Physical activity was determined using a validated physical activity questionnaire [18]. Physical activity comprised the sum of the average hours per week spent in cycling, sports, and gardening during summer and winter. Walking included the sum of the average hours per week during summer and winter.

### 2.4. Blood and Urine Collection and Laboratory Analysis

About 60 mL of venous blood was collected from each participant at the BfR study center. Several routine biomarkers, including serum concentration of alkaline phosphatase, high-sensitivity C-reactive protein (hsCRP), thyroid-stimulating hormone (TSH), zinc, and total homocysteine (using NaF blood), were measured at an accredited medical analytics laboratory (Labor 28 GmbH, Berlin, Germany) on the same day. About half of the blood was fractionated into serum/plasma and erythrocytes and stored at −80 °C until further analysis. In 2018, Labor 28 determined the bone turnover biomarker b-CrossLaps (CTX) and osteocalcin in the serum. Due to an implausibly high value of CTX in a participant, one measurement was not considered in the present study. Serum concentrations of procollagen type-1 (PINP) were measured at Labor Augsburg MVZ GmbH (Augsburg, Germany). Methylmalonic acid and vitamins A, B2, B6, D3, and K1, as well as amino acids alanine, arginine, glutamine, leucine, lysine, and proline were measured in plasma at Bevital AS (Bergen, Norway). Plasma concentrations of fibroblast growth factor 23 (FGF23), α-klotho, and PTH were measured at the Institute of Agricultural and Nutritional Sciences, Martin Luther University Halle-Wittenberg (Halle, Germany). Serum concentrations of holotranscobalamin, vitamin B12, and folate were determined at the Department of Clinical Chemistry and Laboratory Medicine, University of Saarland (Homburg, Germany). Serum concentrations of selenium and selenoprotein P (SePP) were measured at the Institute of Nutritional Science, University of Potsdam (Potsdam, Germany). Fatty acids in plasma phospholipids were determined at the Department of Molecular Epidemiology, German Institute of Human Nutrition Potsdam-Rehbruecke (Germany) [19]. The total n-3 fatty acids included the sum of linolenic acid (C18:3n3), eicosapentaenoic acid (C20:5n3), docosapentaenoic acid (C22:5n3), and docosahexaenoic acid (C22:6n3). We calculated the combined vitamin B12 indicator (4cB12) from concentrations of holotranscobalamin, vitamin B12, total homocysteine, and methylmalonic acid according to the published equation (adjusted for age) [20]. The participants collected their urine over 24 h before their second visit to the study center. Concentrations of calcium and magnesium in 24 h urine were measured at Labor 28 GmbH (Berlin, Germany) and concentrations of urinary iodine at the Laboratory of Human Nutrition, Institute of Food, Nutrition and Health, ETH Zurich (Switzerland) [21].

### 2.5. Sample Size Estimation

The sample size was calculated by assuming a clinically relevant difference of 5% in BUA between vegans and omnivores. Along with a level of significance of 5% and a power of 80%, a total of 72 participants were required (36 vegans, 36 omnivores) (G*power, (*t*-test for independent samples), version 3.1., Heinrich Heine University, Dusseldorf, Germany).

### 2.6. Statistical Analysis

Normally distributed variables are reported as mean ± standard derivation. Skewed variables are reported as median (interquartile range). Categorical variables are reported as *n* (percentages). A Student’s *t*-test or Mann–Whitney *U* test was used to compare the continuous variables between vegans and omnivores, and a chi-square test was used for categorical variables. The RRR was described in detail by Hoffmann et al., including the SAS software code and its application in nutritional epidemiology [22]. RRR appears to be a promising tool for characterizing the relationships between bone health and a comprehensive profile of biomarkers. The RRR determines the linear combinations of predictor variables (biomarkers) that explain a maximum variation in the response variables (BUA and SOS). In this analysis, we used 28 bone-relevant biomarkers as predictor variables. In detail, we included the nutritional biomarkers, i.e., vitamins (combined vitamin B12 indicator, A, B6, B2, K1, folate), amino acids (alanine, arginine, glutamine, leucine, lysine, proline), total n-3 fatty acids, zinc, SePP, urinary magnesium, urinary iodine, TSH, along with biomarkers of calcium homeostasis (PTH, vitamin D3, urinary calcium), biomarkers of bone turnover (CTX, PINP, osteocalcin, alkaline phosphatase), biomarkers of the FGF23–α-klotho axis (α-klotho, FGF23), and the inflammatory biomarker hsCRP. Due to missing values of BUA (*n* = 1) and CTX (*n* = 1), the RRR analysis comprised 70 participants (36 vegans, 34 omnivores). All skewed variables were log-transformed for the analyses. As the number of response variables determines the number of extracted patterns, the current RRR created two patterns. To ensure that the observed variation of bone-relevant biomarkers reflected the different profiles of vegans and omnivores, the RRR patterns were derived using the pooled data of vegans and omnivores. Only the first pattern was retained for the analyses, as this pattern contributed the largest proportion of explained variance (first pattern: 34.4%, second pattern: 5.3%). The description of the bone-health-related pattern focused on those predictors with factor loadings ≥0.20, which were considered the main contributors of a score. Each participant received a factor score for the identified pattern; this score ranked the participants according to the degree to which they conformed to the pattern. Distributions of the main contributors were compared across tertiles of the pattern scores, and analysis of variance (ANOVA) was used to assess the linear trends. Investigating the main contributors across tertiles of the pattern scores, additional analyses were carried out using a multivariable-adjusted analysis of covariance (ANCOVA), including the additional adjustment of the month of assessment (January–July, model 1), a sex- and age-adjusted model 2, as well as a lifestyle model 3 (BMI, smoking status, physical activity, alcohol consumption). Moreover, sensitivity analyses were performed after the exclusion of postmenopausal women (*n* = 6) and one woman with surgical menopause. Linear regression models were used to estimate the associations between diet groups (vegan/omnivores) with BUA (unadjusted, model 1) and adjusted for lifestyle factors (model 2), including age, sex, smoking status, educational level, BMI, physical activity, and alcohol consumption. Models 3 and 4 were adjusted for the biomarker pattern score, while model 4 was additionally for lifestyle factors. All statistical analyses were performed using SAS software, version 9.4 (SAS Institute, Cary, NC, USA). Any *p*-values < 0.05 were considered statistically significant.

## 3. Results

Table 1 shows the basic characteristics of the 72 participants according to a vegan or omnivorous diet (*n* = 36 each). The median duration of veganism was 4.8 years (IQR: 3.1–8.7). Due to sex- and age-matched inclusion of the participants, we observed no differences in sex and age (Table 1). Moreover, no differences in anthropometric measurements, physical activity, smoking, education, or alcohol consumption were observed between the groups (all *p* > 0.05). However, compared to omnivores (33.3%), vegans (97.2%) were more likely to take supplements, especially supplements of vitamin B12 (91.7%).

Compared to omnivores, vegans showed lower mean values of all QUS parameters (Table 2). However, only the difference in BUA levels reached statistical significance (vegans: 111.8 ± 10.7 dB/MHz, omnivores: 118.0 ± 10.8 dB/MHz, *p* = 0.02). In addition, a regression revealed that omnivores had 6.2 dB/MHz higher BUA levels compared to vegans (*p* = 0.02, model 1, Table S1), and the association was even stronger after adjusting for lifestyle factors (model 2, Table S1). The bone resorption marker CTX was higher in vegans (0.45 ± 0.19 ng/mL) compared to omnivorous participants (0.36 ± 0.16 ng/mL, *p* = 0.03). Concerning the calcium homeostasis, vegans had lower urinary calcium levels (*p* = 0.004) and were more likely to have higher PTH concentrations compared to omnivores (*p* = 0.09). Moreover, vegans had higher α-klotho concentrations than omnivores (*p* = 0.01). Omnivores had higher concentrations of vitamin A and B2, whereas vegans showed higher concentrations of vitamin K1 and folate. The concentrations of vitamin B12 and B6 did not differ between the dietary groups. Vegans had higher concentrations of glutamine and lower concentrations of lysine compared to omnivores (*p* < 0.0001, Table 2), whereas there were no differences in the other amino acids (i.e., alanine, arginine, leucine, and proline). Moreover, vegans had a lower level of urinary iodine compared to omnivores (*p* < 0.0001), while the TSH concentration (*p* = 0.34) did not differ. Furthermore, vegans had lower concentrations of zinc (*p* = 0.03), SePP (*p* < 0.0001), and total n-3 fatty acids (*p* < 0.0001).

### Exploratory RRR

**Figure 1:**
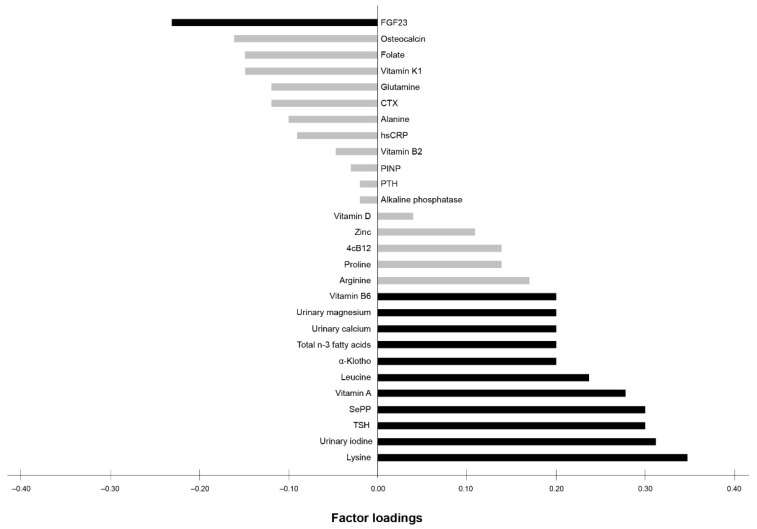
Factor loadings of all 28 biomarkers according to the biomarker pattern score explaining the maximum variation in BUA and SOS. Factor loadings are correlations between biomarkers and the biomarker pattern score. Black bars indicate biomarkers with factor loadings ≥ 0.20, which are considered as major contributors to the score. Grey bars indicate biomarkers with factor loadings < 0.20. FGF23 (fibroblast growth factor 23), CTX (b-CrossLaps), hsCRP (high-sensitivity C-reactive protein), PINP (procollagen type-1), PTH (parathyroid hormone), 4cB12 (four markers combined vitamin B12 indicator), SePP (selenoprotein P), TSH (thyroid-stimulating hormone).

An exploratory RRR was applied to investigate the relationship between bone health (BUA and SOS) and the profile of 28 nutrition- and bone-related biomarkers. The first derived biomarker pattern score explained 34.4% of the total variance in BUA and SOS (35.9% for BUA, 32.9% for SOS). Twelve out of the 28 biomarkers were identified to contribute most to bone health. This pattern consisted of the following main contributors (factor loading of ≥0.20) with positive factor loadings for lysine (0.35), urinary iodine (0.31), TSH (0.30), SePP (0.30), vitamin A (0.28), leucine (0.24), α-klotho (0.20), total n-3 fatty acids (0.20), urinary calcium (0.20), urinary magnesium (0.20), and vitamin B6 (0.20), and negative factor loading for FGF23 (−0.23) (Figure 1).

An ANOVA across tertiles of the biomarker pattern score showed that the levels of all QUS parameters were significantly higher across the tertiles (Table 3). Accordingly, participants in the highest tertile (T3) had, on average, 11.1% higher BUA levels compared to the first tertile (T1) (*p* for trend < 0.0001). Furthermore, we observed an increase in SOS (T1 to T3: 2.6%, *p* for trend < 0.0001), as well as SI (T1 to T3: 18.5%, *p* for trend < 0.0001) across the tertiles of the biomarker pattern score, while the percentage of vegans decreased. In detail, the first tertile comprised 70% vegans, the second tertile had 61% vegans, and the third tertile included 26% vegans (*p* for trend = 0.009). Moreover, across the tertiles, we observed a positive association with physical activity (*p* for trend = 0.01). We observed no association between other lifestyle factors across tertiles (Table 3). Interestingly, a regression model revealed the high impact of the biomarker pattern score on bone health independent of the diet group, as the model detected no difference in BUA between vegans and omnivores after adjustment of the biomarker pattern score (model 3, Table S1).

Regarding the main contributors of the pattern, an ANOVA across tertiles of the biomarker pattern score showed significant positive associations with vitamin A (*p* for trend = 0.003), vitamin B6 (*p* for trend = 0.01), the amino acid lysine (*p* for trend = 0.0002), SePP (*p* for trend = 0.0004), and n-3 fatty acids (*p* for trend = 0.03). Furthermore, participants had higher concentrations of urinary iodine and TSH (both *p* for trend = 0.002) across the tertiles. As depicted in Table 3, according to the FGF23–α-klotho axis, FGF23 concentrations showed inverse associations (*p* for trend = 0.04), whereas α-klotho levels were higher in participants in T3 compared to T1; however, these were not statistically significant across the tertiles (*p* for trend = 0.21). Furthermore, the urinary calcium levels (T1: median 60.0 mg/L vs. T3: 82.0 mg/L), and levels of urinary magnesium (T1: 50.2 mg/L vs. T3: 59.1 mg/L) were higher in participants in T3, although not statistically significant across the tertiles (both *p* for trend > 0.19). Regarding leucine, no association across the tertiles was observed (*p* for trend = 0.14). In addition to the main contributors of the pattern, zinc was positively associated across tertiles (*p* for trend = 0.02, Table S2).

In the sensitivity analyses, after the additional adjustment according to the month of assessment, sex, age, and lifestyle variables, i.e., BMI, smoking status, physical activity, and alcohol consumption, effectively no changes in the results were observed (data not shown). In addition, the exclusion of postmenopausal women and women with surgical menopause did not change the results (data not shown).

## 4. Discussion

The present study observed differences in bone health between vegans and omnivores, showing lower mean values of all QUS parameters in vegans compared to omnivores; however, only differences in the BUA levels reached statistical significance. We also detected differences in biomarkers related to bone health between vegans and omnivores, and an exploratory biomarker pattern was further derived, revealing a combination of biomarkers contributing to bone health. This pattern provides a possible explanation of the lower bone health in vegans compared to omnivores.

Up till now, few studies [7,23,24,25,26,27,28] have investigated the association between a vegan diet and bone health, showing lower BMD in vegans compared to omnivores. In 2019, Iguacel et al. [6] concluded in a systemic review and meta-analysis that a vegan diet was associated with decreased BMD at different sites (lumbar spine, femoral neck, whole body) compared to an omnivorous diet [6]. Moreover, the authors suggested that the lower BMD values found in vegans could be clinically relevant because the fracture risk was also found to be higher in vegans than in omnivores [6]. None of the included studies used QUS data for the assessment of bone health. However, the results of our RBVD study are in agreement, also showing reduced bone health in vegans compared to omnivores.

Scientific evidence suggests that some specific nutrients derived mainly from animal food sources are found in lower quantities in vegans, which could adversely affect bone health. It is well known that vitamin B12 is the most critical nutrient when following a vegan diet [10,12]. Regarding bone health, it has been proposed that a deficiency in vitamin B12 can negatively affect bone development and maintenance [6]. However, we observed no differences in any of the blood parameters assessing vitamin B12 status [21]. Next to vitamin B12, vitamin D also plays a central role in bone metabolism and mineralization. Vitamin D deficiency leads to increased bone turnover, resulting in decreased bone mineral density [29]. Furthermore, Busse et al. assumed that vitamin D deficiency decreases bone turnover and, in turn, leads to premature bone aging [30]. The impaired turnover of vitamin-D-deficient bone leads to hypo- and hypermineralized bone areas and increased fracture risk [30]. Due to the omission of food from animal origins, vegans are at higher risk of inadequate vitamin D supply [10,12,29], which may have adverse bone health effects. Furthermore, the endogenous vitamin D production might be limited in our study population living in Berlin (Germany) due to low sun exposure for several months of the year [29]. However, a sensitivity analysis revealed no change in the results after an adjustment for the month of blood collection. In agreement with the current evidence, the dietary intake of vitamin D3 is lower in vegans [21], but we observed no difference in the vitamin D3 blood concentrations between vegans and omnivores, most likely because 50.0% of our vegans took vitamin D3 supplements.

We detected further differences in nutritional biomarkers between vegans and omnivores, which may contribute to the decreased bone health in vegans. A review of Dai and Koh [13] investigated the possible role of B vitamins in bone health, including evidence from in vitro and in vivo experimental studies, as well as observational and intervention studies. Next to vitamin B12, the results of this review suggest a protective role of vitamins B2 and B6 in bone health [13]. Interestingly, in agreement with the reduced bone health of vegans in the RBVD study, we also observed lower plasma concentrations of vitamin B2 in vegans, which is explained by the lower dietary intake compared to omnivores [21]. Indeed, a few studies have shown that the status of vitamin B2 is considered deficient in ≈30% of vegans [31,32]. Regarding vitamin A, Davey et al. noticed a lower mean intake of retinol in vegans compared to omnivores, fish-eaters, and ovo-lacto-vegetarians in the European Prospective Investigation into Cancer and Nutrition (EPIC)-Oxford study [12]. Although no significant difference in the intake of vitamin A equivalents was observed in the RBVD study [21], the plasma concentrations of vitamin A were lower in vegans compared to omnivores. However, the role of vitamin A regarding bone health may be ambiguous. On the one hand, it has been found that vitamin A promotes skeletal health [33]. On the other hand, an epidemiological study demonstrated that an excessive intake of vitamin A or high serum vitamin A are also related to adverse skeletal health, including accelerating bone loss, decreasing bone mineral density, and increasing the incidence of fractures [33].

As oily fish and, to a lesser extent, dairy foods and meat are the primary sources of eicosapentaenoic acid (EPA) and docosahexaenoic acid (DHA) [34,35], the intake of n-3 fatty acids while following a vegan diet may be lower than in omnivores [10]. Indeed, lower plasma levels of n-3 fatty acids in vegans compared to omnivores were observed in the present study. The n-3 fatty acids EPA and DHA are suggested to stimulate osteoblast survival, promote osteoblastogenesis, and prevent bone resorption by altering membrane function, regulating calcium balance, and enhancing osteoblast activity [36]. Furthermore, the involvement of EPA and DHA in preosteoblast differentiation and maturation was associated with their anti-inflammatory effects, i.e., reducing the synthesis of inflammatory PGE2 and modulating peroxisome proliferators-activated receptor gamma (PPARgamma) and lower levels of inflammatory cytokines, e.g., interleukin-1 (IL-1), interleukin-6 (IL-6), and tumor necrosis factor alpha (TNF-α) [36]. Regarding bone health, a recent meta-analysis on observational studies noticed that a higher dietary intake of n-3 fatty acids was significantly associated with a lower risk of hip fracture [37]. In addition, two systematic reviews/meta-analyses based on randomized controlled trials indicated associations between n-3 fatty acids and improved BMD [38,39].

Different minerals have an impact on bone metabolism. It has been observed that selenium and the selenium-transport protein SePP (constituting the majority of selenium in blood) were positively correlated with BMD [40,41], even if SePP might be more relevant because of its proposed function as the essential selenium transporter to the bones [42]. Vegans had a lower intake of selenium [10], as well as lower concentrations of total serum selenium [41]. In fact, this was also seen in the present study; however, statistical significance was observed only for SePP. Next, zinc has also been found to be important in the regulation of bone homeostasis, as many zinc-related proteins are involved in the regulation of cellular function in osteoblasts and osteoclasts [43]. Zinc stimulates cell differentiation, cell proliferation, and mineralization in osteoblasts [43]. Indeed, a study showed lower BMD for the hip, spine, and distal wrist of men in the lowest plasma zinc quartile compared to men with higher plasma zinc concentrations [44]. Accordingly, the present study demonstrated lower serum zinc concentrations in vegans, as well as lower BUA levels, compared to omnivores. Furthermore, the macro minerals calcium and magnesium are known as important contributors to bone health [43]. In fact, 99% of the body’s calcium resides in the skeleton and about 60% of all magnesium in the body is found in bone [43]. As concentrations in the blood are carefully regulated within narrow limits, the present study used 24 h urine samples to better reflect the calcium and magnesium statuses. A switch from an omnivorous to a vegetarian diet demonstrated a rise in the urinary excretion of magnesium [45]. Kidneys are able to retain magnesium during deprivation by reducing its excretion or excrete magnesium in cases of excess intake [46]. Therefore, the renal excretion of the filtered load has been found to vary from 0.5 to 70% [46]. Nevertheless, the homeostasis also depends on the absorption in the intestine. In fact, it is noteworthy that the intestinal absorption of magnesium is not directly proportional to dietary magnesium intake but is rather dependent on the individual magnesium status [46]. It has been found that the lower the magnesium level, the more this element is absorbed in the gut; thus, relative magnesium absorption is high when intake is low and vice versa [46]. The individual adaption of magnesium might provide a possible explanation for why the present study observed no differences in urinary magnesium concentrations between vegans and omnivores, despite the observed higher intake of magnesium in vegans [12], which is supported by our dietary data. Regarding calcium, a switch from an omnivorous diet to a vegetarian diet is associated with a decrease in the excretion of calcium [45]. In detail, Knurick et al. found that the daily calcium excretion was significantly higher (≈34%) in the omnivores as compared to individuals adhering to vegetarian diets [7]. The present study also showed a lower excretion of calcium in vegans compared to omnivores (≈36%). This was likely caused by the lower intake of calcium in vegans as urinary calcium concentrations reflect dietary intake [47].

A vegan diet may also include healthy constituents that counterbalance the negative effects on bone health. In fact, plant-based diets are high in vitamin K [7,8] and folate [7,12]. Accordingly, our RBVD study demonstrated higher dietary intake [21] and higher concentrations of folate and vitamin K in the blood of vegans compared to omnivores. Vitamin K is known as a cofactor for the optimal mineralization of bone and is positively associated with BMD [11]. In addition, several epidemiologic studies found a significant relationship between high folate intake/concentrations and increased BMD or reduced fracture risk [7,13,14].

Lifestyle factors may influence or cover potential associations between dietary habits and BMD [6]. Scientific evidence suggests that vegans tend to show a healthier lifestyle compared to omnivores, which might have an important impact on BMD [9], i.e., higher levels of physical activity [12], lower smoking rates [12], lower consumption of alcohol [12], and lower BMI. However, as the present study detected no relevant differences in these lifestyle factors between vegans and omnivores, no impact on the levels of QUS measurements was expected.

### Exploratory RRR

As discussed above, several nutrients require particular attention for bone health in vegans. However, ascribing the lower BUA levels (in some degree SOS and SI) of vegans to a single nutrient or biomarker is likely oversimplistic, given the complexity of the homeostatic regulatory mechanisms of bones. In fact, complex interconnections between nutrients, foods, and dietary patterns imply that no single element of a diet can provide the complete picture of dietary effects on health [48]. Based on this, an exploratory systematic approach was adapted to detect a biomarker pattern that revealed a combination of biomarkers that contributes to bone health, i.e., the RRR identified a pattern based on twelve biomarkers as main contributors (factor loading ≥ 0.20) explaining a maximum variation in BUA and SOS in our population. Highly important, the ANOVA demonstrated positive associations between all QUS parameters across the tertiles of the biomarker pattern score. This might be of clinical relevance, as it has been reported that even relatively small changes in bone health, e.g., a 10% increase in bone mass, reduced fracture risk by as much as 50% [9].

The identified biomarker pattern was characterized by biomarkers with positive factor loadings for lysine, urinary iodine, TSH, Sepp, vitamin A, leucine, α-klotho, total n-3 fatty acids, urinary calcium, urinary magnesium, and vitamin B6, and a negative factor loading for FGF23. Regarding the main contributors, the ANOVA supported positive associations of vitamin A and B6, SePP, and n-3 fatty acids across the tertiles of the biomarker pattern score. This is in agreement with the aforementioned recent evidence showing that these biomarkers are suggested to be components with beneficial properties according to bone health [38,39,40,41].

Interestingly, urinary iodine and TSH also seem to have an important role in bone health, identifying them as strong contributors to the biomarker pattern. In fact, a recent epidemiological study reported that urinary iodine levels were significantly lower in women with postmenopausal osteoporosis and were associated with the total T-score [49]. Regarding TSH, a population-based register cohort study that included healthy participants without a known thyroid disease (*n* = 222,138) observed associations between low TSH concentrations with an increased long-term risk of hip fracture (45% increase in hip fracture risk for each SD reduction in TSH level) [50]. Similarly, Murphy et al. also noticed a 43% increase in nonvertebral fracture risk for each SD reduction in TSH levels in 2374 euthyroid postmenopausal women [51].

Furthermore, the RRR also identified the plasma amino acids leucine and lysine as the main contributors to the biomarker pattern. Mechanistic evidence indicated that leucine and lysine (in addition to arginine, alanine, proline, and glutamine) stimulate insulin secretion in vitro [52], which has been proposed to promote osteoblast growth and differentiation [53,54]. Additionally, it has been shown that leucine is the most potent of the branched-chain amino acids for the stimulation of muscle protein synthesis [55], which is critical for the maintenance of adequate bone strength and density [54]. Similarly, Jennings et al. demonstrated that the dietary intake of lysine, leucine (in addition to arginine, alanine, proline, and glutamic acid) was associated with higher BMD [54].

The FGF23–α-klotho axis was also identified as a main contributor to the biomarker pattern. FGF23 was inversely associated. FGF23 plays a key role in balancing mineral ion homeostasis and bone mineralization [56], where it reduces the renal phosphate uptake and the secretion of parathyroid hormone, respectively [57,58]. Moreover, it has been noticed that FGF23 decreases 1,25-dihydroxyvitamin D concentrations by downregulating the expression of vitamin-D-metabolizing enzymes [57,58]. The critical role of FGF23 in mineral ion homeostasis was first identified in human genetic and acquired rachitic disease [56], showing that an excess of FGF23 levels cause several types of hypophosphatemic rickets/osteomalacia, which are characterized by impaired mineralization of the bone matrix [56,57]. This is in agreement with the present study, which found that FGF23 was the biomarker with the strongest negative factor loading in our exploratory RRR. Nevertheless, more research is needed because until now, only a few cross-sectional studies have investigated the association of FGF23 with BMD in apparently healthy participants, providing controversial results [59,60,61,62]. Furthermore, until now, only a few epidemiological studies [60,63,64] have investigated the associations between circulating α-klotho and bone health and showed conflicting results.

To conclude, the exploratory RRR revealed a combination of twelve biomarkers that might have contributed to bone health in our study population. As the present study revealed a decreased percentage of vegans across the tertiles of the biomarker pattern score corresponding with increasing QUS levels, it might be hypothesized that the detected combination of biomarker concentrations contributed to the impaired bone health in vegans. However, as the RBVD was a small study, replication in an independent study population is needed to confirm the results. To the best of our knowledge, the present study is the first to apply RRR to detect an exploratory biomarker pattern that may reveal a combination of biomarkers that are relevant to bone health. Usually, RRR has been efficiently used in nutritional epidemiology to identify dietary patterns [65]. The validation of the derived patterns is highly recommended [65]. Further limitations of our study deserve to be mentioned. In the present study, we used the QUS measurements as a proxy of BMD, commonly measured using the dual energy X-ray absorptiometry technique (DEXA). However, validation studies against DEXA suggested the usefulness of QUS in diagnosing osteoporosis and future fracture risk [66]. Therefore, QUS represents a valid, inexpensive, easy, and quick alternative measurement tool without radiation. Moreover, the cross-sectional design does not allow for causal inference. Moreover, the study included middle-aged men and women from a small area (Berlin, Germany); thus, the results may not be generalizable to other populations. However, the RBVD study provided comprehensive high-quality data as a result of the standardized procedures, including the collection of blood and urine, in combination with extensive information from computer-based questionnaires, a dietary assessment using a 3-day weighed food protocol, and anthropometric measurements.

In conclusion, the study observed differences in bone health between vegans and omnivores, along with differences in biomarkers related to bone health. In addition, an exploratory biomarker pattern was derived that revealed a combination of biomarkers, providing a possible explanation of a reduced bone health in vegans compared to omnivores. Additional studies are required to confirm these findings.

## Figures and Tables

**Table 1:** Characteristics of the study population according to a vegan or omnivorous diet.

Characteristics	Vegans (*n* = 36)	Omnivores (*n* = 36)	*p*-Value
Duration vegan diet (years)	4.8 (3.1–8.7)		
Men	50.0% (18)	50.0% (18)	1.00
Age (years)	37.5 (32.5–44.0)	38.5 (32.0–46.0)	0.75
Anthropometry			
BMI (kg/m^2^)	22.9 ± 3.2	24.0 ± 2.1	0.08
Fat mass (%)	24.1 ± 7.8	26.2 ± 7.7	0.27
Muscle mass (%)	33.9 ± 5.2	33.5 ± 5.2	0.72
Waist circumference (cm)			
Women	73.1 ± 6.9	77.2 ± 6.2	0.07
Men	84.5 ± 8.9	86.0 ± 6.1	0.56
Education (%)			0.60
Low	0.0% (0)	2.8% (1)	
Intermediate	30.6% (11)	30.6% (11)	
High	69.5% (25)	66.7% (24)	
Lifestyle			
Physical activity (h/week)	2.8 (0.88–3.75)	2.3 (1.2–4.1)	0.69
Walking (h/week)	7.0 (5.0–12.0)	5.5 (3.5–11.8)	0.15
Smoking status			0.30
Non-smoker	66.7% (24)	58.3% (21)	
Ex-smoker	22.2% (8)	16.7% (6)	
Smoker	11.1% (4)	25.0% (9)	
Alcohol consumption (g/d)			
Women	0.10 (0.00–4.69)	0.21 (0.02–4.88)	0.22
Men	0.04 (0.00–2.00)	3.85 (0.36–16.2)	0.09
Taking supplements	97.2% (35)	33.3% (12)	<0.0001
Vitamin B12	91.7% (33)	8.3% (3)	<0.0001
Vitamin D3	50.0% (18)	11.1% (4)	0.0003

Variables expressed as percentage (*n*), mean ± SD, or median (IQR). BMI: body mass index.

**Table 2:** Characteristics of bone parameters and biomarkers according to a vegan or omnivorous diet.

Characteristics	Vegans (*n* = 36)	Omnivores (*n* = 36)	*p*-Value
Quantitative ultrasound			
BUA (dB/MHz) ^a^	111.8 ± 10.7	118.0 ± 10.8	0.02
SOS (m/s) ^a^	1581.7 ± 28.3	1592.3 ± 9.27	0.20
SI ^a^	97.3 ± 13.3	104.3 ± 16.9	0.05
Bone turnover			
CTX (ng/mL) ^a^	0.45 ± 0.19	0.36 ± 0.16	0.03
Osteocalcin (ng/mL)	20.8 ± 5.49	18.2 ± 6.83	0.08
PINP (µg/L)	60.7 ± 17.0	52.7 ± 18.2	0.06
Alkaline phosphatase (U/L)	64.5 (57.0–80.0)	59.5 (50.5–79.5)	0.13
Calcium homeostasis			
PTH (pg/mL)	52.3 ± 21.0	44.1 ± 19.0	0.09
Vitamin D3 (nmol/L)	63.2 (21.5–88.1)	45.4 (34.6–68.6)	0.49
Urinary calcium (mg/L)	55.5 (36.5–73.0)	86.0 (49.0–165.5)	0.004
FGF23–α-klotho axis			
α-Klotho (pg/mL)	780.3 (621.1–976.2)	640.1 (520.8–770.2)	0.01
FGF23 (RU/mL)	64.5 (54.4–83.2)	63.6 (57.7–72.5)	0.75
Vitamin B12 status			
Vitamin B12 (pmol/L)	337.9 (218.0–559.1)	267.6 (227.2–364.5)	0.12
Holotranscobalamin (pmol/L)	89.4 (58.9–205.0)	84.3 (67.6–100.4)	0.35
Total homocysteine (µmol/L)	8.60 (6.70–11.3)	8.75 (7.25–10.5)	0.90
Methylmalonic acid (µmol/L)	0.17 (0.15–0.22)	0.18 (0.16–0.21)	0.62
4cB12	0.54 (0.07–1.24)	0.42 (0.19–0.70)	0.47
Vitamins			
Vitamin A (µmol/L)	1.80 (1.56–1.92)	2.07 (1.80–2.33)	0.004
Vitamin B2 (nmol/L)	6.00 (4.39–10.70)	9.05 (6.82–11.8)	0.03
Vitamin B6 (nmol/L)	67.2 (49.1–89.4)	78.8 (47.1–99.7)	0.62
Vitamin K1 (nmol/L)	1.55 (1.30–2.23)	0.78 (0.54–1.13)	<0.0001
Folate (ng/mL)	10.9 (7.71–12.8)	7.82 (6.36–11.2)	0.03
Amino acids			
Alanine (µmol/L)	373.2 ± 98.1	348.7 ± 66.2	0.22
Arginine (µmol/L)	64.1 (52.7–74.4)	69.1 (59.0–76.0)	0.35
Glutamine (µmol/L)	629.4 ± 83.2	546.9 ± 64.3	<0.0001
Leucine (µmol/L)	117.5 (103.6–137.0)	120.0 (114.4–143.8)	0.07
Lysine (µmol/L)	128.5 (119.0–147.7)	171.4 (152.3–189.3)	<0.0001
Proline (µmol/L)	174.7 (146.5–244.4)	174.6 (139.2–195.7)	0.24
Iodine and thyroid			
Urinary iodine (µg/L)	28.1 (17.1–41.6)	74.1 (41.5–101.7)	<0.0001
TSH (µg/L)	2.13 ± 0.92	2.35 ± 1.05	0.34
Other bone-related biomarkers			
Zinc (µg/dL)	79.3 ± 11.6	87.3 ± 13.3	0.008
Selenium (µg/L)	67.7 (59.8–82.1)	76.2 (68.4–83.5)	0.11
SePP (mg/L)	3.26 (2.61–4.47)	4.97 (4.22–5.46)	<0.0001
hsCRP (mg/L)	0.39 (0.21–0.88)	0.63 (0.24–1.74)	0.25
Total n-3 fatty acids (%)	3.07 (2.66–3.53)	5.11 (4.22–5.77)	<0.0001
Urinary magnesium (mg/L)	57.0 (44.8–66.9)	56.4 (43.5–81.9)	0.88

Variables expressed as percentage or mean ± SD or median (IQR); ^a^ *n* = 71 (vegan *n* = 36, omnivores *n* = 35). BUA (ultrasound attenuation), SOS (speed of sound), SI (stiffness index), CTX (b-CrossLaps), PINP (procollagen type-1), PTH (parathyroid hormone), FGF23 (fibroblast growth factor 23), 4cB12 (four markers combined vitamin B12 indicator), TSH (thyroid-stimulating hormone), SePP (selenoprotein P), hsCRP (high-sensitivity C-reactive protein).

**Table 3:** Characteristics of the bone parameters and biomarkers with factor loadings ≥ 0.20 according to tertiles of the first biomarker pattern score obtained using reduced rank regression.

Characteristics	T1 (*n* = 23)	T2 (*n* = 24)	T3 (*n* = 23)	*p* for Trend
Vegans/omnivores	16/7	14/10	6/17	0.009
Duration vegan diet (years)	3.5 (3.1–6.0)	4.9 (2.3–6.3)	8.2 (4.2–12.2)	0.27
Men	39.1% (9)	50.0% (12)	56.5% (13)	0.49
Age (years)	40.0 (35.0–47.0)	36.0 (31.0–44.5)	35.0 (31.0–44.0)	0.09
BMI (kg/m^2^)	22.4 ± 2.5	24.0 ± 3.1	23.7 ± 2.5	0.13
Physical activity (h/week)	1.50 (0.67–3.54)	2.42 (1.07–3.44)	2.67 (1.75–4.33)	0.01
Smoker	26.1% (6)	16.7% (4)	13.0% (3)	0.17
Alcohol consumption (g/d)				
Women	0.27 (0.01–9.90)	0.10 (0.01–2.50)	0.13 (0.02–1.51)	0.16
Men	2.00 (0.21–19.8)	0.03 (0.00–1.99)	1.16 (0.00–4.40)	0.42
Quantitative ultrasound				
BUA (dB/MHz)	108.8 ± 10.8	113.2 ± 9.06	122.4 ± 9.37	<0.0001
SOS (m/s)	1569.1 ± 27.4	1581.5 ± 28.2	1611.7 ± 33.4	<0.0001
SI	91.8 ± 12.9	98.1 ± 12.1	112.7 ± 14.3	<0.0001
Calcium homeostasis				
Urinary calcium (mg/L)	60.0 (39.0–82.0)	55.5 (40.0–103.5)	82.0 (50.0–167.0)	0.20
FGF23–α-klotho axis				
α-Klotho (pg/mL)	666.4 (515.8–865.9)	652.5 (557.8–807.4)	763.0 (689.6–860.4)	0.21
FGF23 (RU/mL)	73.7 (58.9–91.3)	62.6 (57.7–70.9)	63.9 (50.3–78.0)	0.04
Vitamins				
Vitamin A (µmol/L)	1.77 (1.53–1.95)	1.91 (1.61–2.21)	2.04 (1.79–2.31)	0.003
Vitamin B6 (nmol/L)	60.0 (44.1–84.1)	72.3 (46.4–95.0)	84.4 (53.3–126.0)	0.01
Amino acids				
Leucine (µmol/L)	117.7 (106.5–136.8)	118.2 (106.7–137.6)	118.9 (111.7–152.8)	0.14
Lysine (µmol/L)	129.7 (113.9–155.8)	146.3 (128.4–165.8)	166.1 (146.3–187.5)	0.0002
Iodine and thyroid				
Urinary iodine (µg/L)	26.7 (14.8–53.3)	44.6 (29.7–63.2)	70.7 (34.1–103.6)	0.002
TSH (µg/L)	1.75 ± 0.81	2.38 ± 1.12	2.64 ± 0.83	0.002
Other bone-related biomarkers				
SePP (mg/L)	3.37 (2.32–4.77)	3.82 (3.07–5.25)	5.08 (4.15–5.32)	0.0004
Total n-3 fatty acids (%)	3.45 (2.79–4.32)	3.98 (3.02–4.93)	4.36 (3.68–5.65)	0.03
Urinary magnesium (mg/L)	50.2 (44.0–59.0)	59.1 (43.3–93.0)	59.1 (46.6–74.3)	0.19

Variables expressed as a percentage or mean ± SD or median (IQR). BMI (body mass index), BUA (ultrasound attenuation), SOS (speed of sound), SI (stiffness index), FGF23 (fibroblast growth factor 23), TSH (thyroid-stimulating hormone), SePP (selenoprotein P).

## Data Availability

The datasets generated during and/or analyzed during the current RBVD study are not publicly available due to provisions of the written informed consent.

## Supplementary Materials

The following are available online at https://www.mdpi.com/2072-6643/13/2/685/s1, Table S1. Regression models of diet (vegans/omnivores) on broadband ultrasound attenuation (BUA), Table S2. Characteristics of all predictor variables included in the reduced rank regression (RRR) (including those in Table 3) according to the tertiles of the first biomarker pattern score.
