# Supplementary material for: Vegan Diet and Bone Health—Results from the Cross-Sectional RBVD Study
Source: Nutrients. 2021 Feb 21;13(2):685. doi: 10.3390/nu13020685 (PMC7924854; doi:10.3390/nu13020685)
Supplement: Supplementary file 1 [file nutrients-13-00685-s001.pdf]

Table S1. Regression Models of Diet (vegans/omnivores) on BUA

	**Adjustment**	β-coefficient	p-value
**Model 1**	-	6.16	0.02
**Model 2**	Lifestyle factors	7.05	0.006
**Model 3**	Biomarker pattern score	1.89	0.41
**Model 4**	Biomarker pattern score + Lifestyle factors	3.49	0.19

The Regression models used the diet group as predictive variable coded as 0 for vegans and 1 for omnivores. Model 1: unadjusted; Model 2: adjustment of lifestyle factors (age, sex, education, smoking status, BMI, physical activity, alcohol consumption); Model 3: Biomarker pattern score was detected by Reduced Rank Regression (RRR). Model 4: biomarker pattern score and additionally adjustment of lifestyle factors

Table S2. Characteristics of all predictor variables included in the RRR (including those in Table 3) according to the tertiles of the first biomarker pattern score

	T1 (n=23)	T2 (n=24)	T3 (n=23)	p for trend
Vegans/Omnivores	16/7	14/10	6/17	*0.009*
Duration vegan diet (years)	3.5 (3.1-6.0)	4.9 (2.3-6.3)	8.2 (4.2-12.2)	*0.27*
Men	39.1% (9)	50.0% (12)	56.5% (13)	*0.49*
Age (years)	40.0 (35.0-47.0)	36.0 (31.0-44.5)	35.0 (31.0-44.0)	*0.09*
BMI (kg/m^2)	22.4 ± 2.5	24.0 ± 3.1	23.7 ± 2.5	*0.13*
Physical Activity (h/week)	1.50 (0.67-3.54)	2.42 (1.07-3.44)	2.67 (1.75-4.33)	*0.01*
Smoker	26.1% (6)	16.7% (4)	13.0% (3)	*0.17*
Alcohol consumption (g/d)				
Women	0.27 (0.01-9.90)	0.10 (0.01-2.50)	0.13 (0.02-1.51)	*0.16*
Men	2.00 (0.21-19.8)	0.03 (0.00-1.99)	1.16 (0.00-4.40)	*0.42*
**Quantitative ultrasound**				
BUA (dB/MHz)	108.8 ± 10.8	113.2 ± 9.06	122.4 ± 9.37	*<0.0001*
SOS (m/s)	1569.1 ± 27.4	1581.5 ± 28.2	1611.7 ± 33.4	*<0.0001*
SI	91.8 ± 12.9	98.1 ± 12.1	112.7 ± 14.3	*<0.0001*
**Bone turnover**				
CTX (ng/ml)	0.46 ± 0.20	0.42 ± 0.20	0.36 ± 0.13	*0.08*
Osteocalcin (ng/ml)	21.4 ± 7.09	19.1 ± 5.79	18.1 ± 5.99	*0.08*
PINP [µg/l)	60.7 ± 17.4	58.8 ± 17.3	52.1 ± 18.5	*0.10*
Alkaline phosphatase (U/l)	65.0 (58.0-80.0)	59.0 (51.5-77.0)	63.0 (48.0-81.0)	*0.30*
**Calcium homeostasis**				
PTH (pg/ml)	49.6 ± 20.6	45.6 ± 23.1	48.1 ± 17.8	*0.80*

Vitamin D3 (nmol/l)	53.7 (23.1-88.1)	70.7 (37.4-91.4)	40.7 (33.6-68.0)	*0.96*
Urinary calcium (mg/l)	60.0 (39.0-82.0)	55.5 (40.0-103.5)	82.0 (50.0-167.0)	*0.20*
**FGF23-α-klotho-axis**				
α-klotho (pg/ml)	666.4 (515.8-865.9)	652.5 (557.8-807.4)	763.0 (689.6-860.4)	*0.21*
FGF23 (RU/ml)	73.7 (58.9-91.3)	62.6 (57.7-70.9)	63.9 (50.3-78.0)	*0.04*
**Vitamin B12 Status**				
4cB12	0.36 (-0.10-0.70)	0.71 (0.28-1.24)	0.42 (0.17-0.70)	*0.16*
**Vitamins**				
Vitamin A (μmol/l)	1.77 (1.53-1.95)	1.91 (1.61-2.21)	2.04 (1.79-2.31)	*0.003*
Vitamin B2 (nmol/l)	6.46 (4.76-9.86)	10.2 (5.73-14.5)	6.76 (4.24-9.95)	*0.87*
Vitamin B6 (nmol/l)	60.0 (44.1-84.1)	72.3 (46.4-95.0)	84.4 (53.3-126.0)	*0.01*
Vitamin K1 (nmol/l)	1.48 (0.83-1.84)	1.33 (0.82-1.55)	0.87 (0.50-1.67)	*0.05*
Folate (ng/ml)	9.31 (6.54-11.9)	10.9 (7.88-13.4)	7.55 (6.17-11.4)	*0.32*
**Amino acids**				
Alanine (μmol/l)	385.4 ± 106.6	355.2 ± 84.5	348.7 ± 51.8	*0.14*
Arginine (μmol/l)	64.9 (50.9-69.5)	63.8 (53.5-71.7)	71.2 (60.1-79.5)	*0.15*
Glutamine (μmol/l)	623.1 ± 77.2	570.2 ± 86.4	572.0 ± 86.5	*0.04*
Leucine (μmol/l)	117.7 (106.5-136.8)	118.2 (106.7-137.6)	118.9 (111.7-152.8)	*0.14*
Lysine (μmol/l)	129.7 (113.9-155.8)	146.3 (128.4-165.8)	166.1 (146.3-187.5)	*0.0002*
Proline (μmol/l)	172.7 (139.9-212.9)	158.4 (129.8-180.9)	191.7 (160.6-246.5)	*0.55*
**Iodine and thyroid**				
Urinary iodine (μg/l)	26.7 (14.8-53.3)	44.6 (29.7-63.2)	70.7 (34.1-103.6)	*0.002*
TSH (μg/l)	1.75 ± 0.81	2.38 ± 1.12	2.64 ± 0.83	*0.002*
**Other bone related biomarker**				
Zinc (μg/dl)	79.1 ± 11.4	81.8 ± 10.7	87.5 ± 14.7	*0.02*

SePP (mg/l)	3.37 (2.32-4.77)	3.82 (3.07-5.25)	5.08 (4.15-5.32)	*0.0004*
hsCRP (mg/l)	0.47 (0.24-1.98)	0.62 (0.25-1.08)	0.44 (0.17-0.75)	*0.39*
Total n-3 fatty acids (%)	3.45 (2.79-4.32)	3.98 (3.02-4.93)	4.36 (3.68-5.65)	*0.03*
Urinary magnesium (mg/l)	50.2 (44.0-59.0)	59.1 (43.3-93.0)	59.1 (46.6-74.3)	*0.19*

Variables expressed as a percentage or mean ± SD or median (IQR), BMI (body mass index), BUA (ultrasound attenuation), SOS (speed of sound), SI (stiffness index), CTX (b-CrossLaps), PINP (procollagen type-1), PTH (parathyroid hormone), FGF23 (fibroblast growth factor 23), 4cB12 (4 markers combined vitamin B12 indicator), TSH (thyroid-stimulating hormone), SePP (selenoprotein P), hsCRP (high-sensitivity C-reactive protein)
